# Hypoglycemic Efficacy of Rh-aFGF Variants in Treatment of Diabetes in ZDF Rats

**DOI:** 10.3389/fcell.2021.609383

**Published:** 2021-02-18

**Authors:** Li Zhang, Qingde Zhou, Min Chen, Xuanxin Yang, Chao Lu, Wenzhe Sun, Qi Hui, Xiaojie Wang

**Affiliations:** ^1^The First Affiliated Hospital of Wenzhou Medical University, Wenzhou, China; ^2^School of Pharmaceutical Sciences, Wenzhou Medical University, Wenzhou, China

**Keywords:** aFGF, type 2 diabetes, hypoglycemic effect, insulin resistance, safety

## Abstract

Acidic fibroblast growth factor (aFGF) is a promising regulator of glucose with no adverse effects of hypoglycemia. Previous researches revealed that aFGF mediated adipose tissue remodeling and insulin sensitivity. These findings supported rh-aFGF_135_ would be used as a new candidate for the treatment of insulin resistance and type 2 diabetes. In this study, we aimed to investigate the hypoglycemic efficacy of recombinant human acidic fibroblast growth factor 135 (rh-aFGF_135_) with low mitogenic in type 2 diabetic ZDF rats. ZDF rats were treated with rh-aFGF_135_ at a daily dosage of 0.25 and 0.50 mg/kg by tail intravenous injection for 5 weeks. The blood glucose levels, oral glucose tolerance test, insulin tolerance test, HOMA-IR for insulin resistance, serum biochemical parameters, and the histopathological changes of adipose tissue, liver and other organs were detected at designed time point. The glucose uptake activity and anti-insulin resistance effect of rh-aFGF_135_ were also detected in HepG2 cells. Results revealed that rh-aFGF_135_ exhibited a better hypoglycemic effect compared with vehicle group and without the adverse effect of hypoglycemia in ZDF rats. Compared with vehicle group, rh-aFGF_135_ significantly improved the situation of hyperglycemia and insulin resistance. Rh-aFGF_135_ decreased ALT, AST, GSP, and FFA levels noticeably compared with vehicle control group (*P* < 0.01 or *P* < 0.001). After 5 weeks of treatment, high-dosage rh-aFGF_135_ could remodel adipose tissue, and has no influence on other organs. H&E staining showed that rh-aFGF_135_ reduced the size of adipocytes. In addition, rh-aFGF_135_ may improve insulin resistance partly by increasing the protein expression of p-IRS-1 (human Ser 307). As a hypoglycemic drug for long-term treatment, rh-aFGF_135_ would be a potentially safe candidate for the therapy of type 2 diabetes.

## Introduction

In modern society, type 2 diabetes mellitus (T2DM) has turned into a major health problem worldwide as a common metabolic disorder characterized by insulin resistance (Roden, 2012; Canivell and Gomis, 2014; Guariguata et al., 2014). Traditional hypoglycemic agents include biguanides and thiazolidinediones, the former work by reducing glycogen output (directly acting on the liver), and the latter rely on the nuclear receptor peroxisome proliferator-activated receptor γ (PPARγ) to exert the hypoglycemic’s activity (Chang et al., 2013). However, previous studies have revealed that metformin can cause side effects such as liver damage and gastrointestinal discomfort, while thiazolidinediones also lead to weight gain and hypoglycemia (Lehrke and Lazar, 2005). Therefore, development of new anti-diabetic drugs is still a hotspot (Li, 2019).

Acidic fibroblast growth factor (aFGF, also called FGF1), an important member of the fibroblast growth factor family, mainly exists in the kidney and brain tissue and consists of 154 amino acids (Gimenez-Gallego et al., 1985). The role of aFGF is obvious in promoting embryonic development, organogenesis, angiogenesis, and wound healing (Beenken and Mohammadi, 2009; Mohan et al., 2010). Recently, the research published in Nature reported that aFGF would emerge as a new type of insulin sensitizer (Suh et al., 2014). In an earlier study, aFGF was also found to mediate adipose tissue remodeling and insulin sensitivity through PPARγ in aFGF knock-out mice (Jonker et al., 2012). Remarkably, compared with thiazolidinediones traditional sensitizers, rats treated with aFGF by subcutaneous injection have not yet been observed any side effects, such as weight gain or liver steatosis (Chang et al., 2013). In addition, our previous 28-day long-term rh-aFGF_13__5_ toxicity study on the New Zealand rabbits’ damaged skin using Carbomer 940 hydrogel demonstrated that the rh-aFGF_135_ long-term treatment had no obvious adverse effects (Zhang et al., 2020). All of these researches suggest that aFGF would become a potential therapeutic drug to treat type 2 diabetes.

In this study, we aimed to investigate the hypoglycemic efficacy of rh-aFGF variants (rh-aFGF_135_), which consists of 135 amino acids and has much lower mitogenic activity compared with full-length rh-aFGF, in type 2 diabetic ZDF rats. The hypoglycemic effect of rh-aFGF_135_, which had not been previously reported, were evaluated by monitoring the effects on serum glucose levels, oral glucose tolerance test (OGTT), insulin tolerance test (ITT), HOMA-IR for insulin resistance (IR), serum biochemical parameters, and the histopathological changes of organs. And *in vitro*, the hypoglycemic effect of rh-aFGF_135_ was analyzed by detecting the glucose uptake and insulin resistance in HepG2 cells.

## Materials and Methods

### Animals

The mice were housed in a pathogen-free facility at the Tianjin Institute of Pharmaceutical Research, with an ambient temperature of 23 ± 3°C, relative humidity of 55 ± 10%, and 12-h light/12-h dark cycle. All animal experiments were in compliance with the guidelines promulgated by the Tianjin Institute of Pharmaceutical Research (Tianjin, China) and permitted by the Animal Ethical Committee of Tianjin Institute of Pharmaceutical Research (Tianjin, China) to assure humane treatment of all animals used in experiment. Leptin receptor mutation ZDF (fa/fa) rats and ZDF (fa/+) rats were used in the experiments (Charles River Laboratories, Beijing, China). ZDF (fa/fa) rats which can be diet-induced as a type 2 diabetic rat model, were received rh-aFGF_135_ at the dosage of 0.25 or 0.50 mg/kg (Wenzhou Medical University, Zhejiang, China) or saline via tail vein injection daily for 5 weeks, namely low-dosage group (L), high-dosage group (H), or vehicle control group. And ZDF (fa/+) rats with normal blood glucose were injected with saline as normal control.

### Oral Glucose Tolerance Test

To test the ability of rh-aFGF_135_ regulating the blood glucose level, OGTT was performed in the ZDF rats on 26th day. Two hours after intravenous administration, 20% of glucose solution (2.0 g/kg) was administered by intragastric administration to the ZDF rats fasted for 12 h. The concentration of blood glucose was measured by glucometer (Roche, Basel, Switzerland) at 0, 30, 60, 90, 120, 150, and 180 min after oral administration of glucose. A graph showing concentration-time was drawn and the value of area under curve (AUC) was calculated. If the AUC value is significantly lower than the vehicle control, it indicates that rh-aFGF_135_ has improved ZDF rats’ ability to regulate the blood glucose level.

### Insulin Tolerance Test

In order to avoid the possible interferences between the experiments, ITT was performed in the ZDF rats on the 35th day, the day before the end of the experiment. Two hours after intravenous administration of rh-aFGF_135_, ZDF rats fasted for 10 h were intraperitoneally injected with 0.5 IU/kg insulin (Sigma-Aldrich). Subsequently, the levels of glucose were measured by glucometer at 0, 30, 60, 90, 120, 150, and 180 min after insulin injection. The AUC value was obtained in the same way as OGTT. For normal ZDF rats, the insulin administration would decrease the fasted rats’ blood glucose concentration by 50% roughly around 15∼30 min after injection. Around 60–90 min after injection, the fasting blood glucose level should be restored to the normal level. However, if the blood glucose level does not decrease or decreases very little after insulin injection, it indicates that there is insulin resistance in ZDF rats.

### Serum Insulin Measured by ELISA Assay

The levels of fasting blood glucose (FBG) and fasting insulin levels (FINS) were measured with glucometer and commercial ELISA kit (Nanjing Jiancheng Bioengineering Institute, Nanjing, China), respectively. Insulin resistance index was calculated using the homeostasis model assessment-insulin resistance (HOMA-IR) method. HOMA-IR = FINS × FBG/22.5. If the HOMA-IR value is significantly greater than the control group, it indicates that there is insulin resistance.

### Measurement of Serum Biochemical Parameters

At the end of the experiment, the ZDF rats were sacrificed after fasting 12 h. Blood samples were taken from each rat. And serum biochemical parameters were measured with commercial kits as follows: glycosylated serum protein (GSP), high-density lipoprotein (HDL), and low-density lipoprotein (LDL-c) assay kits were purchased from Nanjing Jiancheng Bioengineering Institute (Nanjing, China); aspartate aminotransferase (AST), alanine aminotransferase (ALT), alkaline phosphatase (ALP), and free fatty acid (FFA) assay kits were from Solarbio (Beijing, China).

### H&E Staining

At the end of the study, ZDF rats were fasted for 16 h and sacrificed after anesthesia. The rat liver, kidney, spleen, pancreas, and adipose tissue around the epididymis were removed and fixed in 4% paraformaldehyde solution and made into paraffin tissue blocks with 4 mm thickness. Tissue morphology and overall condition were observed by H&E staining under Nikon upright microscope (Nikon ECLIPSE NI).

### Cell Culture

The human hepatocellular carcinoma cell line HepG2 (Shanghai BioLeaf Biotech Co., Ltd., Shanghai, China) were cultured in DMEM medium (5.5 mM glucose) with 10% FBS, 100 μ/mL penicillin, and 100 μg/mL streptomycin at 37°C in a 5% CO_2_ humidified incubator. The cells were seeded in 96-well or 6-well tissue culture plates cultured until 70 ∼ 80% confluency.

### Cell Viability Assay

Cell viability was measured by MTT assay (Manaharan et al., 2013). HepG2 cells were seeded in 96-well plates at the density of 6 × 10^3^ cells/well (100 μL of medium per well; 6 wells per treatment condition) and incubated overnight at 37°C in a 5% CO_2_ humidified incubator. The cells were then subjected to overnight starvation in serum-free DMEM, washed with PBS to remove non-attached cells and cell debris, and a high glucose-induced damage model was constructed according to the references (Nakagami et al., 2002; Ding et al., 2019). After selecting a glucose concentration of 50 mM to establish the high glucose-induced damage cell model, HepG2 cells were stimulated with or without 25, 100, and 400 ng/mL rh-aFGF_135_ for 24 h at 37°C in a 5% CO_2_ humidified incubator. After that, 20 μL of MTT solution (5.0 mg/mL) was added to each well and incubated for 4 h. Then, the supernatant was discarded, and 120 μL of DMSO was added to each well. After proper mixing, the absorbance at 490 nm was measured using a microplate reader (Molecular Devices, Shanghai, China).

### Measurement of Glucose Uptake Activity on HepG2 Cells

Glucose uptake activity assay of rh-aFGF_135_ *in vitro* was performed essentially as described below. HepG2 cells were seeded in 96-well plates at a density of 6 × 10^3^ cells per well (100 μL of medium per well; 6 wells per treatment condition) and incubated overnight at 37°C in a 5% CO_2_ humidified incubator. After overnight starvation, the cells were stimulated with 3.7, 11.1, 33.3, 100, 300, and 900 ng/mL rh-aFGF_135_ for 24 h. Glucose uptake was measured with a glucose assay kit (Nanjing Jiancheng Bioengineering Institute, Nanjing, China) according to the manufacturer’s protocol. The absorbance at 510 nm was recorded using a microplate reader (Molecular Devices, Shanghai, China), and the glucose consumption rate was calculated.

### Insulin Resistance Model on Cells

Insulin-resistant HepG2 cells were induced with 7.8 nM insulin as described previously (Zhang et al., 2012; Hu et al., 2014; Jiang et al., 2017). The cells were stimulated with or without 25, 100, and 400 ng/mL of rh-aFGF_135_ for 24 h. Glucose uptake was measured with a glucose assay kit according to the manufacturer’s protocol. Absorbance at 510 nm was recorded, and the glucose consumption rate was calculated. If the glucose consumption rate is significantly lower than the control group, it indicates that the cells have developed insulin resistance.

### Western Blotting Analysis

HepG2 cells were seeded in 6-well plates at the density of 3 × 10^5^ cells per well (2 mL of medium per well) and incubated overnight at 37°C in a 5% CO_2_ humidified incubator. The cells were then subjected to overnight starvation in serum-free DMEM, washed with PBS to remove non-attached cells and cell debris. Then we used 7.8 nM insulin to induce insulin resistance model as described previously, and the cells were stimulated with 400 ng/mL rh-aFGF_135_ or 0.5 mM metformin (positive control) for 1 h. After that, HepG2 cells were lysed with the RIPA buffer with protease inhibitor, phosphatase inhibitor and PMSF. After centrifugation at 12,000 *g* and 4°C for 15 min, the supernatants were collected. The protein concentrations were quantified via BCA protein assay kit (Beyotime Institute of Biotechnology, Shanghai, China). Then, equal amount of protein was separated via 12% SDS-PAGE gel electrophoresis and transferred to a 0.22 μm polyvinylidene difluoride (PVDF) membrane. Subsequently, the PVDF membranes were blocked with 5% skimmed milk (BD/DicoTM, NJ, United States) in tris-buffered saline tween-20 (TBST) for 90 min at room temperature and incubated with the following antibodies overnight at 4°C. The source and dilution of each antibody are as follows: anti-pIRS-1 (human Ser 307) (1:300) was purchased from Santa Cruz Biotechnology; anti-IRS-1 (1:1000) was from SAB (MD, United States), and anti-GAPDH (1:5000) was from Proteintech (Wuhan, China). The membranes were washed four times and then incubated with horseradish peroxidase (HRP)-conjugated goat anti-rabbit or anti-mouse IgG (1:5000) at room temperature for 45 min. Subsequently, the PVDF membranes were washed three times with TBST, after which signals were detected by an electrochemiluminescence (ECL) chemiluminescent agent, and the results were captured via a ChemiDoc XRS + Imaging System (Bio-Rad, Munich, Germany). Finally, the gray values of the target bands were analyzed by the Image Lab software.

### Statistical Analysis

Statistical analysis of quantifiable results was performed by GraphPad Prism 5 software using *t*-test and ANOVA analysis. All statistics are expressed as mean ± standard deviation (SD). A value of *P* < 0.05 indicates statistical significance.

## Results

### Rh-aFGF_135_ Improves Cell Viability and Glucose Uptake Activity of HepG2 Cells

**FIGURE 1:**
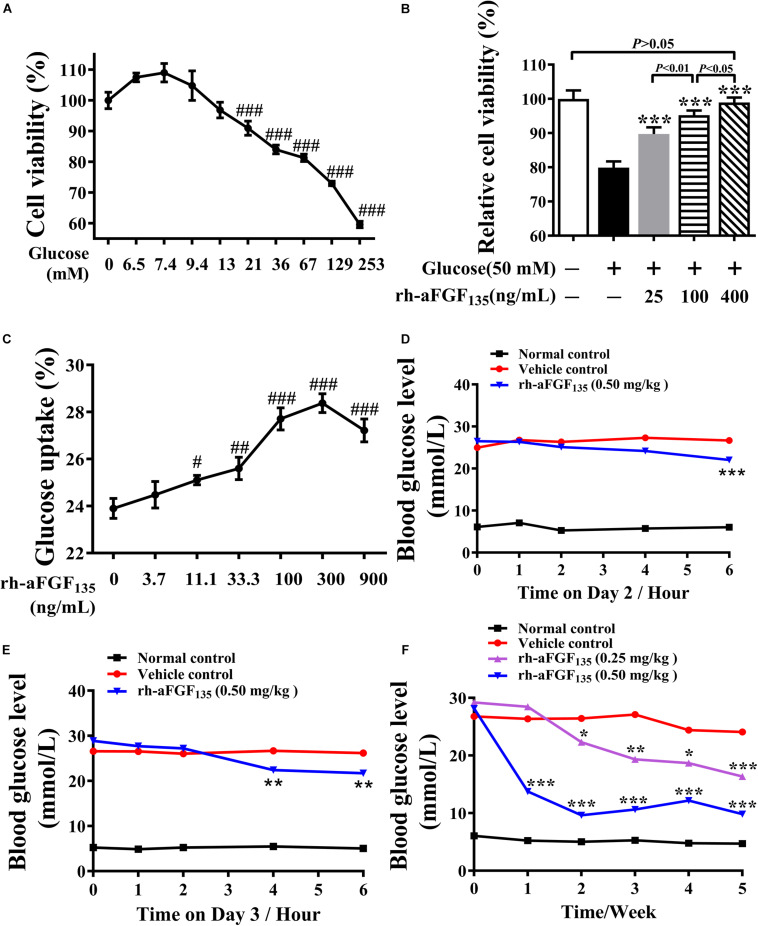
Effects of rh-aFGF_135_ on cell viability and glucose uptake activity, and the anti-diabetic effect of rh-aFGF_135_ in diabetic ZDF rats. **(A)** HepG2 cell viability under different concentrations of glucose in glucose-induced damage model. **(B)** The effect of rh-aFGF_135_ on HepG2 cell viability in 50 mM glucose-induced damage model. **(C)** Glucose uptake rate of HepG2 cells after treatment with different concentrations of rh-aFGF_135_. **(D–F)** The blood glucose level of ZDF rats on the second day **(D)**, and the third day **(E)** and within 5 weeks **(F)** after rh-aFGF_135_ administration. Compared to normal control, ^###^*P* < 0.001, ^##^*P* < 0.01, ^#^*P* < 0.05; Compared to vehicle control, ***P < 0.001, **P < 0.01, *P < 0.05, *n* = 6.

In this study, we tested whether rh-aFGF_135_ could improve HepG2 cell viability in high glucose condition. Cell viability was evaluated by MTT assay. HepG2 cells viability was affected significantly by glucose concentration. As glucose concentration exceeded 21 mM, the cell viability decreased obviously (Figure 1A, *P* < 0.001). In the high glucose-induced damage model caused by 50 mM glucose, when HepG2 cells were stimulated with 25, 100, and 400 ng/mL rh-aFGF_135_ for 24 h, the cell viability improved significantly in a dose-dependent manner compared with high glucose-induced damage group (Figure 1B, *P* < 0.001). Subsequently, we used different concentrations of rh-aFGF_135_ to detect the glucose uptake activity in HepG2 cells. Results showed that rh-aFGF_135_ could significantly improve the glucose consumption rate on HepG2 cells in a dose-dependent manner compared with the control group from 11 to 300 ng/mL (Figure 1C).

### Rh-aFGF_135_ Shows Hypoglycemic Effect in ZDF Rats

In the present study, we used ZDF rats to examine the hypoglycemic effect of rh-aFGF_135_ injection intravenously once a day for 5 weeks. We found that on the second day, 6 h after the administration, the blood glucose level dropped by 17.6% in the high-dosage rh-aFGF_135_ group (Figure 1D, *P* < 0.001). On the third day, the same blood glucose level was achieved only 4 h after the administration (Figure 1E, *P* < 0.01). After treatment for 1 week, high-dosage rh-aFGF_135_ group exhibited a better hypoglycemic effect compared with vehicle group (12.5 ± 3.9 vs. 26.4 ± 2.3 mmol/L, *P* < 0.001). Up to the second week, the blood glucose levels in low-dosage rh-aFGF_135_ group also showed significant differences compared with the vehicle group (22.7 ± 1.9 vs. 26.4 ± 1.6 mmol/L, *P* < 0.05). After treatments for 5 weeks, the blood glucose level of the rats treated with high-dosage rh-aFGF_135_ was close to that of normal rats and did not cause hypoglycemia (Figure 1F). These results revealed that rh-aFGF_135_ play a better hypoglycemic effect in a dose-dependent manner in ZDF rats.

### Rh-aFGF_135_ Improves the Tolerance of Glucose in ZDF Rats

**FIGURE 2:**
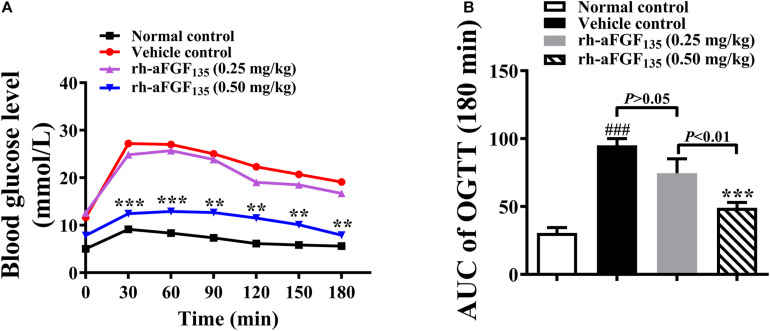
Effect of rh-aFGF_135_ on oral glucose tolerance in diabetic ZDF rats. **(A)** Blood glucose levels in OGTT. **(B)** AUC value of OGTT. Compared to normal control, ^###^*P* < 0.001; Compared to vehicle control, ***P < 0.001, **P < 0.01, *n* = 6.

After treatment with rh-aFGF_135_ for 26 days, when rh-aFGF_135_ could exert a stable hypoglycemic effect, OGTT was performed. The graph of concentration-time was drawn and the value of AUC was calculated and analyzed. The blood glucose levels in high-dosage rh-aFGF_135_ group were significantly lower than those in the vehicle group after taking oral glucose from 0 to 180 min (Figure 2A, *P* < 0.01 or *P* < 0.001). ZDF rats treated with rh-aFGF_135_ showed enhanced glycemia control, while the vehicle group lost the ability to control blood glucose, so the starting blood glucose remained at the high level. The AUC value of vehicle group was significantly higher than the normal group. However, compared with the vehicle control group, AUC value of rh-aFGF_135_-treated group was reduced in a dose-dependent manner (Figure 2B, *P* < 0.001). Altogether, these results indicated that rh-aFGF_135_ can improve the tolerance of glucose in ZDF rats.

### Rh-aFGF_135_ Ameliorates Insulin Resistance in ZDF Rats and HepG2 Cells

**FIGURE 3:**
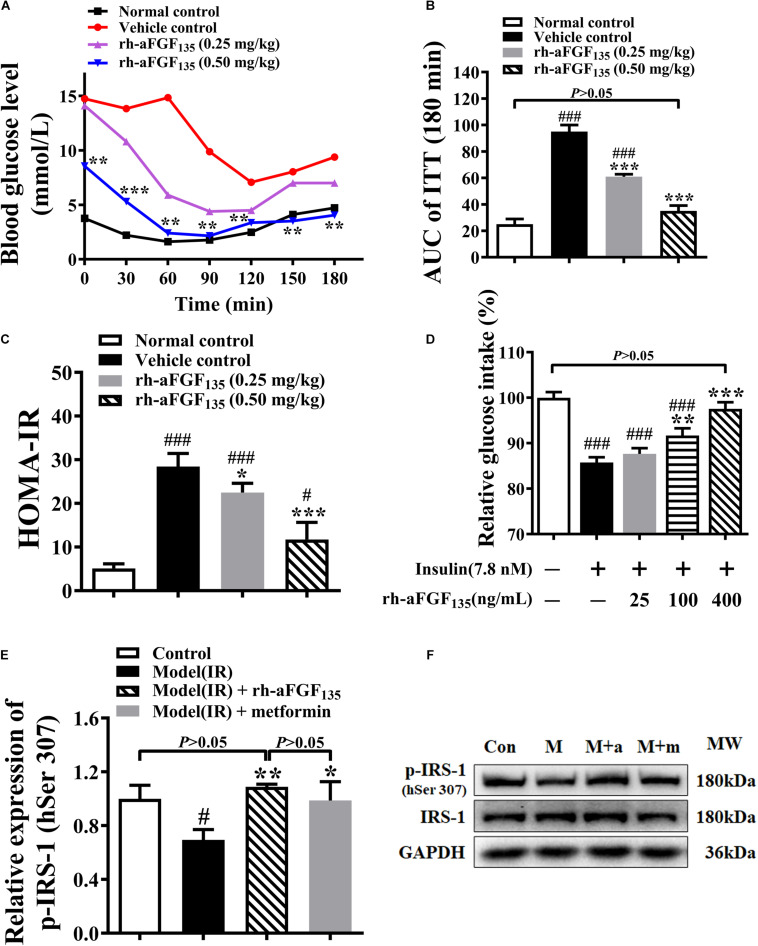
Effects of rh-aFGF_135_ on insulin resistance in diabetic ZDF rats and HepG2 cells. **(A)** Blood glucose levels in ITT in ZDF rats. **(B)** AUC value of ITT in ZDF rats. **(C)** HOMA-IR in ZDF rats. **(D)** Effect of rh-aFGF_135_ on insulin resistance model in HepG2 cells. **(E)** Statistical analysis of relative expression of p-IRS-1 (human Ser 307, hSer 307) in HepG2 cells. **(F)** Western blotting of p-IRS-1 (human Ser 307, hSer 307) and IRS-1, Con: Control; M: Insulin resistance model; M + a: Insulin resistance model and rh-aFGF_135_ (400 ng/mL); M + m: Insulin resistance model and metformin (0.5 mM, positive control). Compared to normal control, ^###^*P* < 0.001, ^#^*P* < 0.05; Compared to vehicle control, ***P < 0.001, **P < 0.01, *P < 0.05, *n* = 6.

At the end of the experiment, in order to observe whether rh-aFGF_135_ could ameliorate insulin resistance in ZDF rats, ITT was analyzed. The results of ITT indicated that the blood glucose levels of rh-aFGF_135_ groups continued to decrease within 60 ∼ 90 min (Figure 3A). The AUC value of rh-aFGF_135_ groups was lower than that in the vehicle control group (Figure 3B, *P* < 0.001). ZDF rats are characterized by insulin resistance thus the levels of serum insulin were investigated. There were no significant differences in serum insulin concentration between all groups (data not shown). However, HOMA-IR values of rh-aFGF_135_ groups were significantly lower than that in the vehicle control group, due to much lower blood glucose levels (Figure 3C, *P* < 0.05 or *P* < 0.001).

The effect of rh-aFGF_135_ on insulin resistance was also examined in HepG2 cells. Compared with control group, 7.8 nM insulin induced insulin resistance and inhibited the absorption of glucose in HepG2 cells. When HepG2 cells were stimulated with 100 and 400 ng/mL rh-aFGF_135_, insulin resistance was improved and glucose uptake was promoted significantly (Figure 3D, *P* < 0.01 or *P* < 0.001), and there was no significant difference between the 400 ng/mL rh-aFGF_135_ treatment group and the control group in terms of glucose uptake rate. In order to detect whether aFGF improved insulin resistance on HepG2 cells by affecting the insulin signaling pathway, we detected the phosphorylation of insulin receptor substrate-1 (p-IRS-1) on serine 307 of human origin by western blotting. The results showed that rh-aFGF_135_ significantly increased the p-IRS-1 (human serine 307) in the insulin-resistant HepG2 cells (Figures 3E,F, *P* < 0.01 or *P* < 0.05). These findings indicated that rh-aFGF_135_ could ameliorate the insulin resistance in ZDF rats and HepG2 cells.

### Rh-aFGF_135_ Partially Improves Serum Biochemical Parameters of ZDF Rats

**FIGURE 4:**
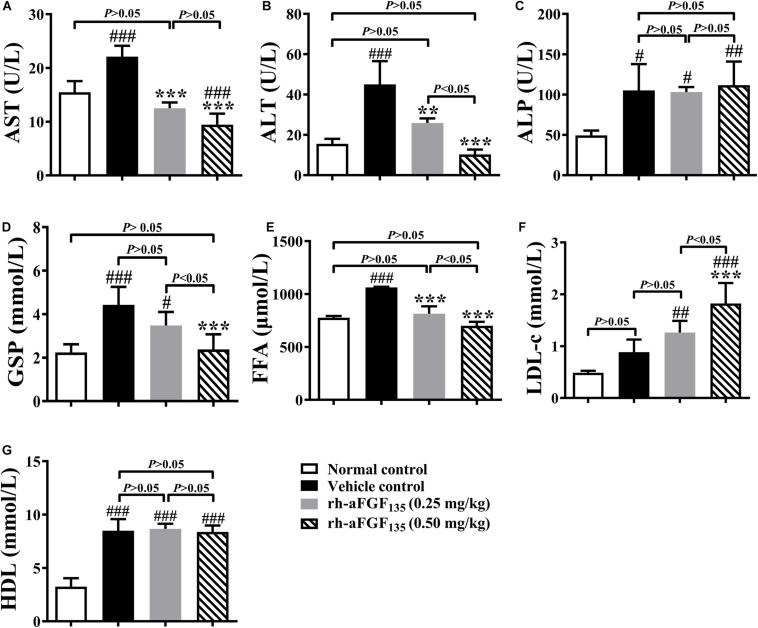
Effects of rh-aFGF_135_ on AST **(A)**, ALT **(B)**, ALP **(C)**, GSP **(D)**, FFA **(E)**, LDL-c **(F)**, and HDL **(G)** levels in the serum of diabetic ZDF rats. Compared to normal control, ^###^*P* < 0.001, ^##^*P* < 0.01, ^#^*P* < 0.05; Compared to vehicle control, ***P < 0.001, **P < 0.01, *n* = 6.

At the end of the experiment, ZDF rats were sacrificed after fasting 12 h. The levels of AST, ALT, ALP, GSP, FFA, LDL-c, and HDL were measured. The data showed that low-dosage and/or high-dosage rh-aFGF_135_ decreased AST, ALT, GSP, and FFA levels significantly compared with vehicle control group (Figure 4, *P* < 0.01 or *P* < 0.001). High-dosage rh-aFGF_135_ could significantly increase the level of serum LDL-c (Figure 4F, *P* < 0.001). However, the levels of ALP and HDL did not differ from the vehicle control group (Figures 4C,G, *P* > 0.05).

### Rh-aFGF_135_ Remodels Adipose Tissue Without Any Side Effects on Other Tissues

**FIGURE 5:**
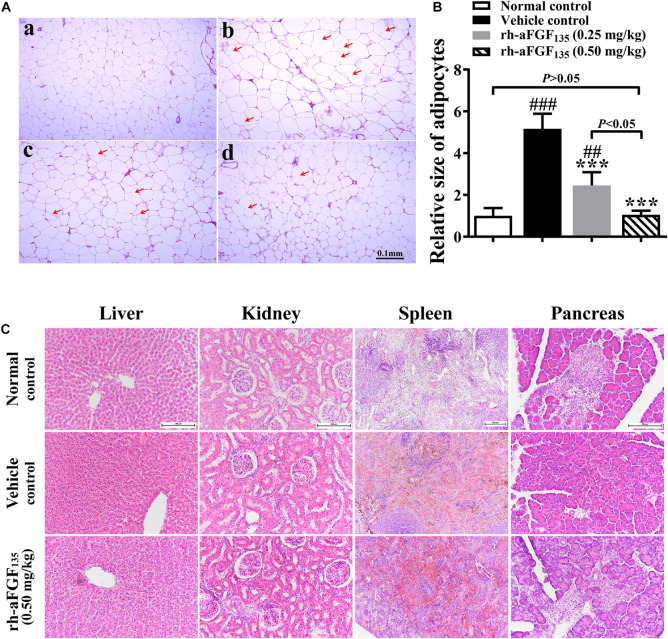
Effects of rh-aFGF_135_ on the organs of diabetic ZDF rats. **(A)** The effect of rh-aFGF_135_ on the pathology of adipose tissue (100×) in diabetic ZDF rats (H&E staining). **(a)** Normal group. **(b)** ZDF rats treated with vehicle. **(c)** ZDF rats treated with rh-aFGF_135_ (0.25 mg/kg). **(d)** ZDF rats treated with rh-aFGF_135_ (0.50 mg/kg). The dead adipocytes were marked with red arrows. **(B)** Statistical analysis of relative size of adipocytes. **(C)** H&E staining of liver (200×), kidney (200×), spleen (100×), and pancreas (200×) in diabetic ZDF rats treated with rh-aFGF_135_. Compared to normal control, ^###^*P* < 0.001, ^##^*P* < 0.01; Compared to vehicle control, ***P < 0.001, *n* = 6.

Some researches demonstrated that obesity-related adipocyte degeneration causes release of cell-free DNA (cf DNA), which promoted macrophage accumulation in adipose tissue and induced insulin resistance of adipose tissue (Bartels et al., 2020). In addition, fat storage capacity is also affected by the size of adipocytes. In this study, we investigated adipose tissue morphology by H&E staining. Compared with the vehicle group, rh-aFGF_135_ significantly reduced the size of adipocytes in a dose-dependent manner (Figures 5A,B, *P* < 0.001). In the vehicle group, there was some visible dead adipocytes. Whereas, high-dosage rh-aFGF_135_ significantly improved the degeneration of adipocytes (marked with red arrows), and has no influence on other organs (Figure 5C). These findings revealed that rh-aFGF_135_ may be involved in adipose tissue remodeling.

## Discussion

Type 2 diabetes mellitus is a chronic and complex metabolic disorder disease that is characterized by hyperglycemia resulting from insulin resistance (Steppan et al., 2001). Although, there is an array of anti-diabetic drugs to cure T2DM but most of them are accompanied by side effects such as weight gain, hypoglycemia, and so on. Recently, some researches revealed the function of aFGF on regulating glucose and lipid metabolism which made it a new drug to cure T2DM (Suh et al., 2014; Li, 2019). In this study, we used diabetic ZDF rats to investigate the pharmacological effects of rh-aFGF_135_ in the treatment of type 2 diabetes.

Controlling blood glucose level is one of the most important indexes of hypoglycemic drugs, so we studied the effect of rh-aFGF_135_ on blood glucose levels of diabetic ZDF (fa/fa) rats (Nwose et al., 2006). In addition, we determined the physiological effects such as OGTT, ITT, HOMA-IR, and serum biochemical parameters in ZDF rats. In this study, the blood glucose levels of the treatment groups were decreased compared with vehicle control group. The OGTT further proved that rh-aFGF_135_ could significantly improve the ability of ZDF rats to regulate blood glucose. Insulin resistance is one of the central causes in the development of type 2 diabetes and was revealed by IR value (Stickel and Hellerbrand, 2010). ZDF rats are characterized by insulin resistance, so we measured the serum insulin levels at the end of the whole experiment and found that there were no significant differences between all groups (data not shown), indicating that rh-aFGF_135_ didn’t stimulate insulin secretion. This is consistent with the previous study which reported that parenteral aFGF might act as an insulin sensitizer rather than insulin secretagogue or insulin mimetic (Suh et al., 2014). The result of HOMA-IR showed that rh-aFGF_135_ could ameliorate insulin resistance in ZDF rats. Remarkably, rh-aFGF_135_ did not lead to excessive hypoglycemia. We also obtained similar results *in vitro* experiments, which was rh-aFGF_135_ could protect HepG2 cells from damage caused by high glucose and alleviated insulin resistance. Many studies have reported that phosphorylation of IRS-1 at serine 307 of human origin (hSer307) is closely related to the enhancement of insulin signal transduction (Giraud et al., 2004; Danielsson et al., 2005; Copps and White, 2012). And our results from western blotting suggested that rh-aFGF_135_ regulate insulin resistance partially by activating phosphorylation of IRS-1 (human Ser 307) in HepG2 cells.

ALT and AST are two biomarkers of liver damage, which are associated with hyperglycemia in diabetes (Postic et al., 2004; Goorden et al., 2013). The ALT and AST levels in rh-FGF_135_-treated groups decreased obviously, indicating that rh-aFGF_135_ may alleviate hepatocyte injury in ZDF rats. In addition, rh-aFGF_135_ revealed its role in adipose remodeling in a dose-dependent manner. Pathological hypertrophy of adipocytes is one of the features of diabetes. Previous studies have shown that small adipocytes are more sensitive to insulin and have better fat storage capacity than hypertrophic adipose tissue (Lundgren et al., 2007). Our results showed that rh-aFGF_135_ could significantly reduce the size of adipocytes in the adipose tissue of ZDF rats, indicating that rh-aFGF_135_ could increase the sensitivity of adipocytes to insulin and enhance the fat storage capacity of adipocytes. Through the above results, we found that rh-aFGF_135_ revealed the ability to regulate blood glucose and improved the insulin sensitivity in ZDF rats.

Our study shows that rh-aFGF_135_ could be used as a candidate for diabetes treatment with no side effect of hypoglycemia which would have great advantages in some special diseases. For example, patients with stress diabetes are often accompanied by symptoms like coma. The ordinary hypoglycemic drugs cannot prevent hypoglycemia while ensuring rapid blood glucose reduction. However, it won’t be a problem for rh-aFGF_135_ because it can ensure no hypoglycemia as we found in our experiments. Although our experiments pointed out that the hypoglycemic effect of rh-aFGF_135_ may be related to the activation of IRS-1 (human Ser 307), its pathways and mechanisms should be further studied.

In conclusion, rh-aFGF_135_ can decrease serum glucose level, ameliorate insulin resistance and reduce the adipocyte size. Rh-aFGF_135_ featured a hypoglycemic effect with no side effect of hypoglycemia. In some special case, rh-aFGF_135_ may be a potential therapeutic agent for its hypoglycemic effect.

## Data Availability Statement

The original contributions presented in the study are included in the article/supplementary material, further inquiries can be directed to the corresponding author.

## Ethics Statement

The animal study was reviewed and approved by the Animal Ethical Committee of Tianjin Institute of Pharmaceutical Research.

## Author Contributions

LZ, QZ, and MC carried out the studies and collected the data. XY and CL performed the statistical analysis and participated in its design. WS and QH participated in acquisition and analysis of data. XW designed the study and wrote the manuscript. All authors have read and approved the final manuscript.

## Conflict of Interest

The authors declare that the research was conducted in the absence of any commercial or financial relationships that could be construed as a potential conflict of interest.

## Abbreviations

ALT: Alanine aminotransferase
ALP: alkaline phosphatase
AUC: area under curve
AST: aspartate aminotransferase
BCA: bicinchoninic acid
cfDNA: cell-free DNA
DMEM: Dulbecco’s modified eagle medium
DMSO: dimethyl sulfoxide
ECL: electrochemiluminescence
ELISA: enzyme linked immunosorbent assay
FBG: fasting blood glucose
FINS: fasting insulin
FBS: fetal bovine serum
FFA: free fatty acid
GAPDH: glyceraldehyde-3-phosphate dehydrogenase
GSP: glycosylated serum protein
H&E staining: hematoxylin-eosin staining
HDL: high-density lipoprotein
HOMA-IR: homeostasis model assessment-insulin resistance
HRP: horseradish peroxidase
IRS-1: insulin receptor substrate-1
IR: insulin resistance
ITT: insulin tolerance test
IU: international unit
LDL-c: low-density lipoprotein-cholesterol
MTT: 3-(4,5-dimethylthiazol-2-yl)-2,5-diphenyltetrazolium bromide
OGTT: oral glucose tolerance test
PPARγ: peroxisome proliferator-activated receptor γ
PMSF: phenylmethyl sulfonylfluoride
PBS: phosphate buffer saline
p-IRS-1: phospho-insulin receptor substrate-1
PVDF: polyvinylidene difluoride
RIPA: radio immunoprecipitation assay
rh-aFGF_135_: recombinant human acidic fibroblast growth factor 135
SD: standard deviation
SDS-PAGE: sodium dodecyl sulfate-polyacrylamide gel electrophoresis
TBST: tris buffered saline tween
T2DM: type 2 diabetes mellitus
ZDF rats: Zucker diabetic fatty rats.
